# Supplementary material for: “I abandoned my job to look after my baby.” Understanding the unpriced cost of care of a preterm infant: Caregivers’ lived experiences
Source: PLoS One. 2023 Aug 17;18(8):e0290101. doi: 10.1371/journal.pone.0290101 (PMC10434847; doi:10.1371/journal.pone.0290101)
Supplement: S1 Appendix — (DOCX) [file pone.0290101.s001.docx]

SUPPLEMENT 1: **Guide used during the focus group discussions and in-depth interviews on experience and utilization of long-term follow-up services for children preterm at Mulago National Referral Hospital.**

Participants: Parents of preterm babies who attended the preterm clinic at Mulago Hospital.

Key characteristics of the mothers: Age, Group Description: Young/Old Mothers, Completed/ Did not complete follow-up for at least one year.

1. In your view, who is a preterm infant?
2. Do you know any complications affecting these infants? **Mention some.**
3. What has been your experience taking care of a preterm infant?

Prompts

- In comparison with your other children, especially born at term
- Cost of care
- Working after birth of a preterm baby

1. What is the importance of following up preterm infants? How long should they be followed up and why?
2. Do you know of any mothers who had their babies followed up for one year or longer? What do you think enabled them to have this follow up?
3. Do you know of any mothers who were not able to have their babies followed up for less than one year? What are the likely reasons they were not able to bring their babies for follow-up?

**Probe in line with:**

➢ Infant factors: Baby well/stable so need for follow-up, baby had grown

➢ Caregiver factors: Not important since nothing is done, baby will another relative who could not bring him to the clinic.

➢ Socio-economic status: No transport fare, no family support, had to take care of other children, had to return to work.

➢ Health system factors: Clinic very far from home, Clinic too crowded, few health workers, rude HCWs, no medicines in the clinic, I took baby near my home or to another facility, HCW told me baby was okay and no need for further follow-up.

1. What should be done to ensure that all preterm babies attend and complete long-term follow-up for at least one year?

**Probe for**

▪ Infant

▪ Caregiver

▪ Health systems factors

1. Do you have any other comment?

**Thank you for your active participation?**
