# Supplementary material for: “I abandoned my job to look after my baby.” Understanding the unpriced cost of care of a preterm infant: Caregivers’ lived experiences
Source: PLoS One. 2023 Aug 17;18(8):e0290101. doi: 10.1371/journal.pone.0290101 (PMC10434847; doi:10.1371/journal.pone.0290101)
Supplement: Attachment — Submitted filename: comments from the AE.docx [file pone.0290101.s002.docx]

Abstract:

Line 3: what other costs are you referring to, if they are not direct

Line 12 -13: i) complex nature of the infant, ii) time to care for the infant, iii) mother as the primary caregiver. The phrasing of these findings, does not make them sound like indirect costs that you set out to explore.

Introduction:

This section is not focused on the challenge at hand – indirect costs associated with care for newborns after discharge from the hospital.

1. The problem/gap in knowledge is not clearly articulated in this section.
2. What have other researchers reported on this subject of indirect costs in low resource settings, beyond mentioning that it is estimated to be 10 times higher than in hospital care.
3. Please revise this to fous on; what do we know about this issue, what don’t we know about it(gap)?, and what do you want to do about it? (aim of your study)

Methods:

One of the key things missing in this section is the lack of a conceptual/theoretical frame work that has informed this analysis. Relatedly, the Equator guidelines for reporting qualitative research were not followed, this makes this section very uninformative and missing a lot of relevant information.

Results;

In table 1- please provide an informative and descriptive title that can make it stand alone.

– complete the table margins as well.

Discussion

1. The key message in this section is difficult to discern in the current state, it is probably related to the misalignment between the title, aim and results reported. What should be the take home message for the readers?
2. What theoretical framework underpins the discussion of your results?
3. Line 280 – it should be cost and not course. Relatedly, there are many typos and grammatical errors all over the document that you need to revise.

Conclusion:

Line 295- “provides understating of the unpriced “hidden” costs caregivers experience in context”. I believe that this wording might be a better way to state your aim and title of the study given the results being reported, as opposed to “indirect costs”.

Reference:

1. Number 6 – 11 are incomplete
2. Please check al the other references to ensure that they conform to the journal guidelines.
